# Anisotropic Strain Relaxation in Semipolar $\left(11\bar{2}2\right)$ InGaN/GaN Superlattice Relaxed Templates

**DOI:** 10.3390/nano12173007

**Published:** 2022-08-30

**Authors:** Wenlong Li, Lianshan Wang, Ruohao Chai, Ling Wen, Zhen Wang, Wangguo Guo, Huanhua Wang, Shaoyan Yang

**Affiliations:** 1 Key Laboratory of Semiconductor Materials Science, Beijing Key Laboratory of Low Dimensional Semiconductor Materials and Device, Institute of Semiconductors, Chinese Academy of Sciences, Beijing 100083, China; 2 College of Materials Science and Opto-Electronic Technology, University of Chinese Academy of Sciences, Beijing 101408, China; 3 Institute of High Energy Physics, Chinese Academy of Sciences, Beijing 100049, China

**Keywords:** semipolar (1122) GaN, InGaN/GaN superlattice templates, strain relaxation, MOCVD

## Abstract

Semipolar (11$\bar{2}$2) InGaN/GaN superlattice templates with different periodical InGaN layer thicknesses were grown on m-plane sapphire substrates using metal-organic chemical vapor deposition (MOCVD). The strain in the superlattice layers, the relaxation mechanism and the influence of the strain relaxation on the semipolar superlattice template were explored. The results demonstrated that the strain in the (11$\bar{2}$2) InGaN/GaN superlattice templates was anisotropic and increased with increasing InGaN thickness. The strain relaxation in the InGaN/GaN superlattice templates was related to the formation of one-dimension misfit dislocation arrays in the superlattice structure, which caused tilts in the superlattice layer. Whereas, the rate of increase of the strain became slower with increasing InGaN thickness and new misfit dislocations emerged, which damaged the quality of the superlattice relaxed templates. The strain relaxation in the superlattice structure improved the surface microtopography and increased the incorporation of indium in the InGaN epitaxial layers.

## 1. Introduction

Light-emitting diodes (LEDs) and laser diodes (LDs) based on the wurtzite InGaN/GaN materials system have been extensively commercialized in many fields, such as solid-state lighting, display, optoelectronic devices and visible light communications [1,2,3]. GaN epitaxial films that are grown along semipolar and nonpolar directions could effectively suppress the quantum confined Stark effect (QCSE) that is caused by spontaneous polarization and piezoelectricity polarization along the traditional c-plane direction, and they have been widely investigated for the fabrication of green or longer emission wavelength GaN-based devices [4,5,6,7]. InGaN/GaN multiple quantum wells (MQWs) along a semipolar orientation have a higher indium incorporation rate than those along a nonpolar orientation, and they are preferable candidates for long wavelength LEDs and LDs [8,9]. However, in the long wavelength region, the mismatch of lattice constants between the InGaN well and the underlying GaN layer is remarkable, and a significant compressive stress is induced in the InGaN quantum wells (QWs), which causes defects and a deterioration in performance.

Stress engineering plays a critical role in the design of high-performance GaN-based devices [10]. A strain-relaxation InGaN buffer underlayer can be introduced to decrease the lattice mismatch in the InGaN/GaN active region, which could also regulate the wavelengths of the InGaN/GaN QWs by increasing indium incorporation [11,12]. Previous results showed that the growth of the multiple quantum wells (MQWs) on the relaxed thick InGaN layers could effectively improve electroluminescence (EL) intensity, decrease the full width at half maximum (FWHM) of the EL and be used to fabricate high-performance (11$\bar{2}$2) LEDs [13,14]. However, the misfit dislocations (MDs), which would be introduced during the heteroepitaxial growth of the InGaN buffer underlayer on the GaN layer when the epilayer thickness exceeded the Matthews–Blakeslee critical thickness, would also affect the performance of the device [15]. It was found that the strain relaxation mechanisms of the underlayer of the InGaN layer also affected the performance of the device. Strain relaxation in semipolar (11$\bar{2}$2) InGaN heteroepitaxial films most likely occurs due to glide of threading dislocations on the (0001) basal slip operation, with the direction parallel to the m-axis to relieve the resolved shear stresses, which forms one-dimensional (1D) MD arrays and causes an epilayer lattice tilt along the c-projection [16,17]. It has also been reported that the relaxation process is related to the combined effects of the non-basal slip operation and the basal slip operation, which forms two-dimensional (2D) MD arrays, causing the epilayer lattice tilt along the c-projection and the m-azimuth; this would seriously damage the device performance [18]. In addition, the preparation of a high-quality relaxed thick InGaN underlayer that has a high In content is extremely challenging due to the solid-phase miscibility gap, which results in phase separation and surface roughening of the InGaN epitaxial layer [19,20]. The researchers found that the thick InGaN film quality could be effectively improved by periodically inserting GaN interlayers that reduce the effect of surface indium accumulation and improve the quality of the InGaN film [21,22]. However, stress in semipolar or nonpolar GaN heteroepitaxial films is expected to be anisotropic due to lattice mismatch and the thermal expansion coefficients along different directions, which results in the distortion of the basal plane of the hexagonal unit cell, making the strain-relaxation analyses in semipolar GaN heteroepitaxial films more complex than those in polar GaN heteroepitaxial films [23,24,25,26]. The research on stress relaxation in semipolar structures is crucial for the strain modulation, structure design and the growth of high-quality semipolar (11$\bar{2}$2) relaxed templates; however, there is still very little research on quantitative strain relaxation analysis in semipolar (11$\bar{2}$2) InGaN/GaN superlattice templates.

In this work, semipolar (11$\bar{2}$2) InGaN/GaN superlattice templates with varying periodic InGaN layer thicknesses were grown on m-sapphire substrates. The strain relaxation and strain anisotropy in these superlattice structures were explored. Additionally, we analyzed the strain relaxation mechanisms in InGaN/GaN superlattice templates and analyzed the influence of strain relaxation on the quality of the superlattice, strain relaxation, surface microtopography and indium incorporation concentration. This research could help in understanding strain relaxation and its effects in semipolar (11$\bar{2}$2) InGaN/GaN superlattice structures, which would be beneficial for the design of high-performance semipolar (11$\bar{2}$2) GaN-based devices.

## 2. Materials and Methods

**Figure 1:**
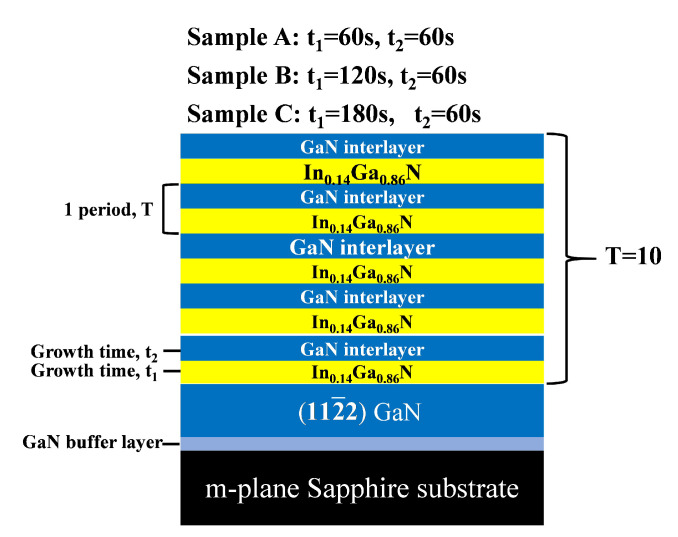
Schematic diagram of the In${}_{0.14}$Ga${}_{0.86}$N/GaN superlattice structures.

Semi-polar (11$\bar{2}$2) GaN superlattice templates were grown on (10$\bar{1}$1) m-plane sapphire substrates in a low-pressure metal-organic chemical vapor deposition (MOCVD) reactor (AIXTRON 200/4 RF-S). Trimethyl gallium (TMGa) and ammonia (NH ${}_{3}$) were used as gallium and nitrogen sources, respectively. At the beginning, the sapphire substrates were thermally annealed in ambient hydrogen at 1130 °C and 100 mbar for 10 min to remove surface contamination. They were then nitridated by introducing NH ${}_{3}$ at 1130 °C for 10 min. Low-temperature (LT) GaN buffer layers were then deposited at 545 °C and 200 mbar for 5.5 min and thick GaN layers were grown at 1130 °C and 60 mbar for 120 min in ambient hydrogen. Finally, as shown in Figure 1, 10 period InGaN/GaN superlattice layers with InGaN layers of different thicknesses were grown on the GaN layers at 780 °C, and the growth time of the interlayer GaN was 60 s and the growth time of the periodic InGaN layers were 60 s for Sample A, 120 s for Sample B and 180 s for Sample C. The In incorporation concentration in the In${}_{x}$Ga${}_{1-x}$ N layers was approximately 0.14. The microtopography and roughness of the samples were measured using an atomic force microscope (AFM, Veeco D3100). The crystal quality and structural properties of these templates were measured by high-resolution X-ray diffraction (HRXRD) using a Huber five-circle diffractometer at the Diffuse X-ray Scattering Station of Beijing Synchrotron Radiation Facility. The radiation energy of the X-ray beam was 8.05 keV with a wavelength of 1.54573 Å. Reciprocal space mapping (RSM) was further measured along the on-axis and the off-axis (11$\bar{2}$2) reflection planes to analyze the strain in the superlattice structures. The photoluminescence spectrums were measured using a Raman spectrometer (LabRAM HR Evolution) with a 325 nm laser unit.

## 3. Results

**Figure 2:**
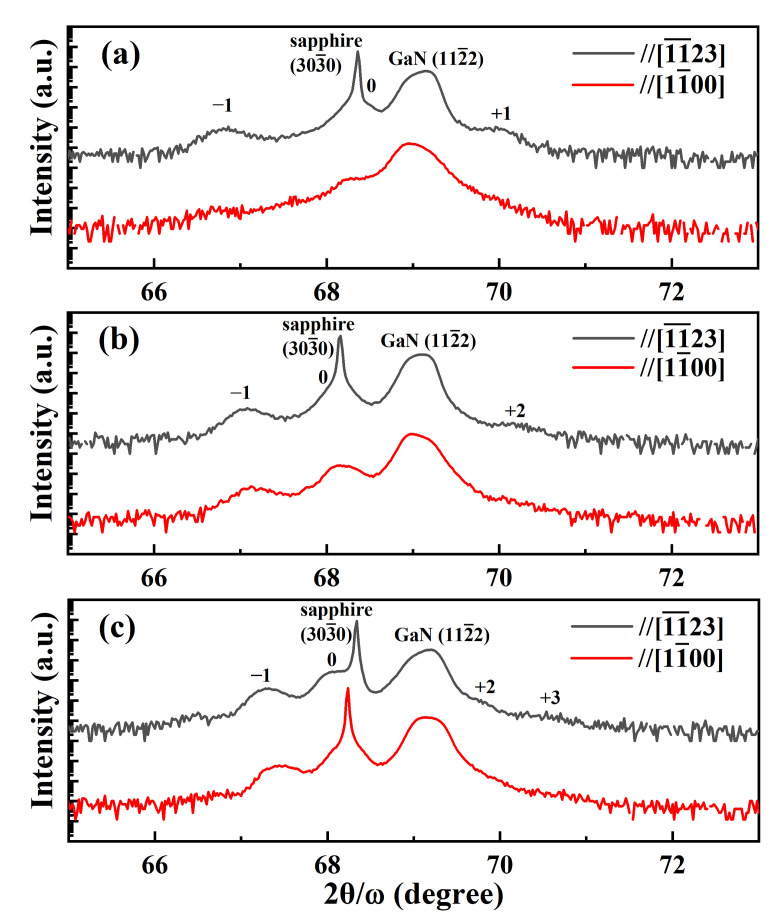
The 2θ/ω scan profiles of the symmetric (11$\bar{2}$2) plane of the superlattice structures along the [1$\bar{1}$00] and the [$\bar{11}$23] directions: (**a**) Sample A; (**b**) Sample B; (**c**) Sample C.

Figure 2 shows the high-resolution x-ray diffraction (XRD) symmetric 2θ/ω scans of the different (11$\bar{2}$2) GaN templates along the [1$\bar{1}$00] and the [$\bar{11}$23] directions. The main peak was related to the thick (11$\bar{2}$2) GaN layer, and the peaks at the shoulder originated from the InGaN/GaN superlattice structure. Satellite diffraction peaks could be observed in different superlattice templates, and the distance between the 0th and −1th satellite diffraction peaks enlarged, indicating the increasing thicknesses of the InGaN layers in the periodic InGaN/GaN structure. Furthermore, the intensity of the satellite peaks enhanced with the increasing thicknesses of the InGaN layers in the InGaN/GaN superlattice templates, which demonstrated the better InGaN/GaN periodic superlattice structure for the thicker InGaN layer.

The XRD reciprocal space maps (RSMs) are a powerful means, which can not only be applied to calculate the structure parameter and analyze the strain relaxation of the epitaxial layers but also to identify the crystallographic layer tilt by analyzing whether the reciprocal lattice point (RLP) of the epitaxial layers is situated on the line connecting the origin with the RLP of the substrate [27,28]. For example, when the semipolar superlattice templates are in a fully strained status, the RLPs of the epitaxial layers of any in-plane wurtzite lattice constant should be coincident and along a line from the origin. In addition, a series of satellite peaks of different diffraction orders lying parallel to the modulation in reciprocal space would be observed. The position of the zeroth-order satellite peak and the distance between the different diffraction order satellite peaks depend on the mean strains and the structure parameter in the superlattice structure. The period thickness (T${}_{SL}$ = t${}_{InGaN}$ + t${}_{GaN}$) of the superlattice templates can be calculated by the following equation (Equation (1)) [29,30]:

(1) $$T_{SL}=2\pi \frac{n_{i}-n_{j}}{Q_{zi}-Q_{zj}},$$

where $n_{i}$ and $n_{j}$ are the orders of the satellite peaks, and $Q_{zi}$ and $Q_{zj}$ are the reciprocal space coordinates of the corresponding peak.

**Figure 3:**
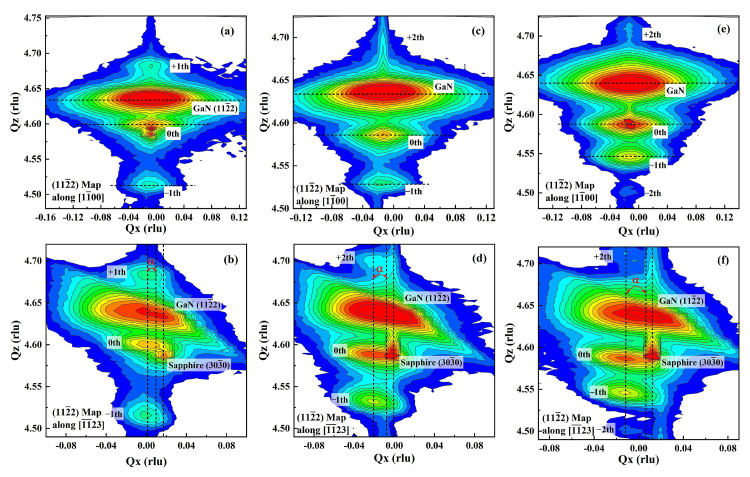
Reciprocal space maps of the (11$\bar{2}$2) plane of superlattice templates along the [1$\bar{1}$00] and the [$\bar{11}$23] directions: (**a**,**b**) Sample A; (**c**,**d**) Sample B; (**e**,**f**) Sample C.

**Figure A1:**
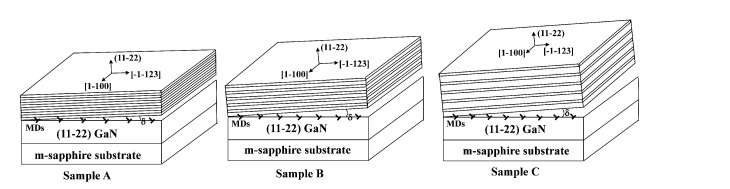
Schematic views of different InGaN/GaN superlattice epilayers grown on semipolar (11$\bar{2}$2) GaN showing the misfit dislocations and tilting angles.

Figure 3 shows the RSMs of the symmetric on-axis (11$\bar{2}$2) GaN reflections along both the [$\bar{11}$23] and the [1$\bar{1}$00] in-plane directions for Sample A, Sample B and Sample C. As shown in Figure 3a,c,e, when the (11$\bar{2}$2) GaN RSMs are measured along the [1$\bar{1}$00] direction, the reciprocal lattice points (RLPs) of the m-plane sapphire, the GaN underlayer and the superlattice layers for these templates were nearly in alignment along the Q ${}_{x}$ direction, and no offset was observed, indicating that the InGaN/GaN superlattice layers were fully strained and coherently grown on the (11$\bar{2}$2) GaN layers along the [1$\bar{1}$00] direction. However, along the [$\bar{11}$23] direction, the RLPs of the m-plane sapphire, the GaN underlayer and the superlattice layers showed a significant offset. The tilt angle (∼0.06°–0.07°) between thet m-plane sapphire and the GaN underlayer could be observed for all the templates. The relative RLPs offset between the InGaN/GaN superlattice layers and the GaN underlayer for the superlattice templates along the [$\bar{11}$23] direction enlarged with increasing InGaN thickness, which demonstrated the increasing strain relaxation. The epitaxial tilt angle, δ, was used to analyze the strain relaxation degree, and the results showed that the tilt angle for the semipolar (11$\bar{2}$2) superlattice increased with increasing periodic InGaN thickness. The observed epitaxial tilts in the semipolar superlattice templates were highly anisotropic along the two in-plane directions so that there was significant tilting (up to δ = 0.11° for Sample A, δ = 0.22° for Sample B and δ = 0.62° for Sample C) parallel to the [$\bar{11}$23] direction, and minimal tilting (∼0° for all the samples) parallel to the [1$\bar{1}$00] direction, which demonstrated that the strain relaxation mechanism for these superlattice templates could be related to the formation of one-dimensional MD arrays [18,31]. For wurtzite III-nitride heterostructures grown on semipolar polar [32,33], the most favorable slip system is the (0001) basal plane with slip directions of b = 1/3[11$\bar{2}$0]-type and the emergence of shear stresses on the inclined (0001) c-plane at heterointerfaces, which lead to the formation of misfit dislocations (MDs) with line directions along [1$\bar{1}$00] and Burgers vectors that are parallel to the [11$\bar{2}$0] direction. The existence of MDs release the resolved shear stress induced by the glide of pre-existing threading dislocations (TDs) on the (0001) plane and results in the inclination of the epitaxial superlattice layers with respect to the underneath (11$\bar{2}$2) GaN layer. A simple equation is used to estimate the relationship between MDs and tilts: δ = b ${}_{edge,⊥}$/MD spacing = b ${}_{edge,⊥}$·$\rho_{MD}$, where b${}_{edge,⊥}$ = b sin θ, θ is the inclination angle of the (11$\bar{2}$2) plane with respect to the (0001) plane and b is the Burgers vector [31,34,35]. The detailed results are summarized in Table 1. The increasing epitaxial tilts and strain-relaxation in the superlattice templates were due to the increased MD density in the superlattice layers, as shown in Figure A1. In addition, the RLP intensity of the InGaN/GaN superlattice layers was stronger with increasing InGaN thickness, indicating the improved crystal quality of the superlattice layers.

To further analyze strain relaxation in superlattice structures, it is essential to calculate the lattice parameters of the underlying GaN layer and the superlattice layer; however, the presence of anisotropic in-plane strain in semipolar GaN epitaxial layers distorts the basal plane of the hexagonal unit cell, resulting in the lattice parameters (*a*, *c*, α, β and γ), which deviate from the ideal hexagonal crystal values.It is very complicated to obtain precise lattice parameters of semipolar epitaxial films and a series of reflections in skew-symmetric and (11$\bar{2}$2) in symmetric geometry [26] need to be measured. Due to the complexity of the superlattice structure in the semipolar directions and the limitation of experimental conditions, we have to simplify the analysis method. For simplifying the calculation process, we assumed that the anisotropic strain would alter the lattice parameters (*a*, *c* and γ), and the lattice parameters (α and β) were still 90°. Therefore, the interplanar distance $d_{\left(hkl\right)}$ was approximately expressed by the lattice parameters (*a*, *c* and β) as

(2) $$\frac{1}{d_{hkl}^{2}}=\frac{h^{2}+k^{2}-2hkcos\gamma }{{\left(asin\gamma \right)}^{2}}+\frac{l^{2}}{c^{2}}$$

where *a*, *c* and γ represent the distorted semipolar unit cell. Based on the above equation, the asymmetric RSMs of the (11$\bar{2}$0), (20$\bar{2}$0) and (10$\bar{1}$1) reflections of the superlattice templates were also measured to precisely calculate the lattice parameters. The interplanar spacing is $d_{hkl}=2\pi /\sqrt{Q_{x}^{2}+Q_{y}^{2}}$, where the in-plane and out-of plane components of the reciprocal lattice vector could be obtained from the RSMs of the on-axis and the off-axis (11$\bar{2}$2) reflections [36]. We then calculated the lattice parameters of the semipolar GaN layer and the zeroth-order satellite peak (SL${}_{\circ }$) of the superlattice layers, which represented the mean lattice parameters of the superlattice structure shown in Table 2. Additionally, we compared the experimental interplanar spacing of the (10$\bar{1}$1) planes with the calculated values obtained from the obtained lattice parameters, and the error was less than 1%, demonstrating that the simplified model had good accuracy. With the lattice parameters derived from Table 2, the out-of plane and the in-plane strain between the semipolar GaN underlayer and the superlattice layers can be deduced from the equations below.

Out-of plane strain:

(3) $$\varepsilon_{(11\bar{2}2})=\frac{d_{\left(11\bar{2}2\right)}^{SL_{0}}-d_{\left(11\bar{2}2\right)}^{GaN}}{d_{\left(11\bar{2}2\right)}^{GaN}}$$

In-plane strain:

(4) $$\varepsilon_{\left[\bar{11}23\right]}=\frac{L_{\left[\bar{11}23\right]}^{SL_{0}}-L_{\left[\bar{11}23\right]}^{GaN}}{L_{\left[\bar{11}23\right]}^{GaN}}$$

(5) $$\varepsilon_{\left[1\bar{1}00\right]}=\frac{L_{\left[1\bar{1}00\right]}^{SL_{0}}-L_{\left[1\bar{1}00\right]}^{GaN}}{L_{\left[1\bar{1}00\right]}^{GaN}}$$

where *d* and *L* are the interplanar spacing and distance, respectively; index “*SL${}_{0}$*” and “*GaN*” denote the superlattice layer and the GaN underlayer, respectively. The calculated results of the in-plane and out-of plane strains for different superlattice templates are summarized in Table 3. As shown in Table 3 it is noticeable that the strains in the semipolar (11$\bar{2}$2) plane along the [$\bar{11}$23] direction and the [$1\bar{1}$00] direction for all samples were anisotropic, and the in-plane and out-of plane strains of superlattice templates were larger for thicker InGaN thicknesses, indicating the increasing strain relaxation in the superlattice layers. Additionally, the increasing tendency of strain in the superlattice layers with the increasing InGaN thickness became slower, and the in-plane strain along the [$1\bar{1}$00] direction emerged, demonstrating that new misfit dislocations would be introduced, which could cause the epitaxial films to tilt along the m-azimuth direction [18]. Therefore, an appropriate InGaN layer thickness was significant to relieve stress and obtain high-quality semipolar (11$\bar{2}$2) InGaN/GaN superlattice relaxed templates.

**Figure 4:**
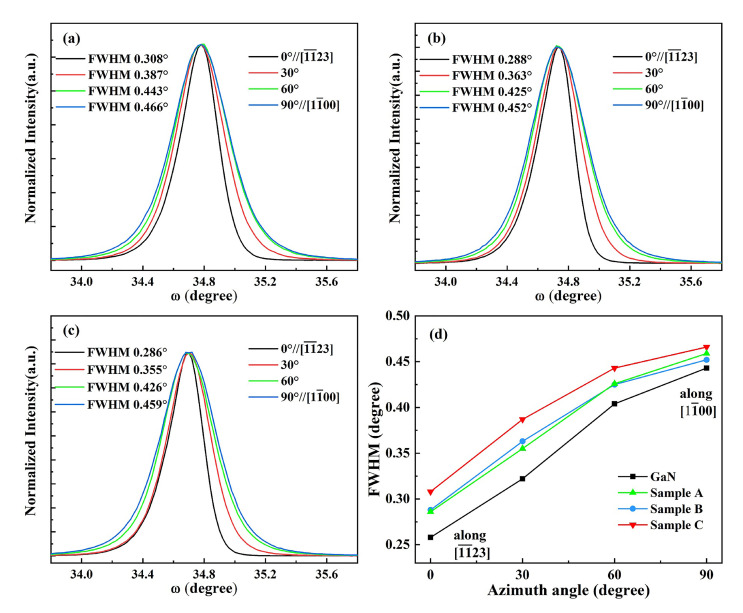
The XRC results of the (11$\bar{2}$2) GaN with respect to different azimuthal angles in different superlattice structures: (**a**) Sample A; (**b**) Sample B; (**c**) Sample C; (**d**) the variation trend of FWHM for different structures.

To further explore the influence of strain relaxation on the crystalline quality of superlattice templates, we measured the X-ray rocking curves (XRCs) of the (11$\bar{2}$2) GaN superlattice templates with respect to different azimuthal angles, where ϕ = 0° and 90° represent the projection of the incident X-ray beam that is parallel to the [$\bar{11}$23] direction and the [$1\bar{1}$00] direction, respectively. As shown in Figure 4, the XRCs of the symmetric (11$\bar{2}$2) GaN reflections for all the (11$\bar{2}$2) GaN superlattice templates showed anisotropy and broadened with the increasing azimuthal angles. These could have been caused by the difference in the surface striations, the distribution of defects, such as dislocations and stacking faults (SFs), as well as a mosaic tilt or a different coherent length (the size of the mosaic blocks) along the [$\bar{11}$23] direction and the [$1\bar{1}$00] direction and have already been observed in nonpolar or semipolar GaN films. We found that the full width at half maximum (FWHM) values of the XRCs of the (11$\bar{2}$2) GaN reflection for superlattice templates increased slightly with the increasing periodic InGaN thickness and were larger than that without the superlattice structures, which were mainly attributed to the increasing misfit defects in the strain-relaxation superlattice templates. Additionally, the variation trend of the XRCs FWHMs for the superlattice templates along the [$\bar{11}$23] direction was more remarkable than those along the [$1\bar{1}$00] direction, which might also demonstrate anisotropic strain relaxation in the semipolar (11$\bar{2}$2) superlattice templates.

**Figure 5:**
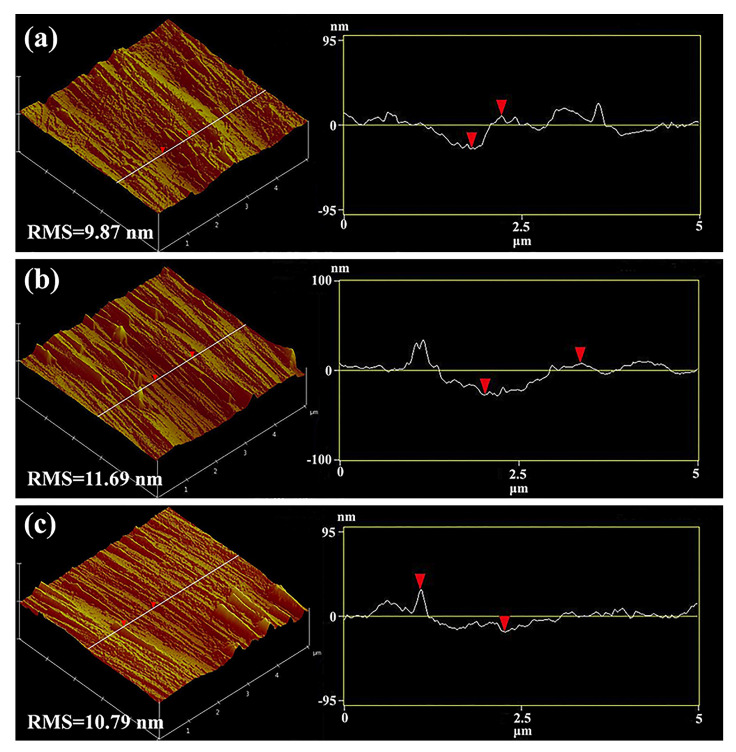
AFM 3D-microtopography and height profiles along the white line of the (11$\bar{2}$2) GaN templates with different superlattice structures: (**a**) Sample A; (**b**) Sample B; (**c**) Sample C.

Figure 5 shows the 5×5 μm^2^ AFM 3D microtopographic images of the (11$\bar{2}$2) GaN superlattice templates. A typical crosshatch surface morphology that showed a characteristic undulating morphology with hills and valleys parallel to the [$\bar{11}$23] direction for the (11$\bar{2}$2) GaN superlattice templates could be remarkably observed, which was directly correlated with the presence of an interfacial misfit dislocation [37,38]. As the InGaN thickness increased in the superlattice structures, the strain energy increased and an additional misfit dislocation formed at the heterogenous interface to relieve the resulting strain in the remarkable crosshatch surface morphology. The root mean square (RMS) roughnesses of these three samples were 9.87 nm, 11.69 nm and 10.79 nm, respectively. Although Sample A presented a smaller RMS surface roughness, the surface microtopographic appeared uneven, and the height profile was very undulating, which might be against the following growth of the MQWs. However, as the thickness of the InGaN layer increased, the microtopographic was effectively improved, demonstrating that strain relaxation is beneficial to realize a better microtopography for the fabrication of (11$\bar{2}$2) GaN superlattice templates.

**Figure 6:**
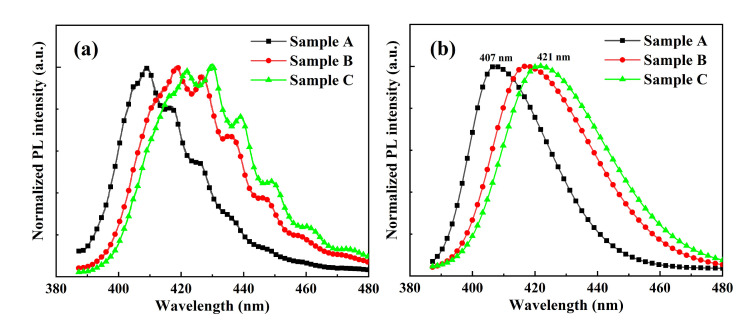
Photoluminescence spectrums of Sample A, Sample B and Sample C at room temperature: (**a**) experimental data; (**b**) fitting curves.

Figure 6 shows the experimental and fitting photoluminescence (PL) spectra of different superlattice templates at room temperature. Redshift could be observed with increasing strain relaxation and the emission peaks decreased from 407 nm for Sample A to 421 nm for Sample C, indicating the increasing indium incorporation concentration. According to the PL spectrum results, we estimated the indium composition in the InGaN/GaN superlattice templates based on the empirical formula [39]. The calculated indium compositions for Sample A, Sample B and Sample C were 9.4%, 11.3%, and 12.3%, respectively. The PL FWHMs of Sample A, Sample B and Sample C were 25 nm, 31 nm, and 32 nm, respectively, which showed an increasing tendency with increasing strain relaxation. The discrepancy of the FWHMs between the fully-strained template and strain-relaxation template could be related to the difference of indium incorporation along the growth direction due to the composition pulling effect.

## 4. Conclusions

In summary, we prepared semipolar (11$\bar{2}$2) InGaN/GaN superlattice templates with different periodical InGaN layer thicknesses on m-sapphire substrates. The strain in the semipolar (11$\bar{2}$2) superlattice layers and the influence of strain relaxation on the semipolar superlattice template were analyzed in detail. The results showed that the strain in the (11$\bar{2}$2) InGaN/GaN superlattice layers was anisotropic and the strain relaxation increased with increasing InGaN thickness. Additionally, strain relaxation occurred through the formation of one-dimension MD arrays at the heterostructure of the superlattice layer and underneath the GaN layer, which caused the tilts in the superlattice structure with respect to the (11$\bar{2}$2) GaN growth direction. However, with increasing strain, the in-plane strain along the [1$\bar{1}$00] direction emerged and new misfit dislocations were introduced, which caused the epitaxial films to tilt along the m-azimuth direction and impaired the performances of the devices. Therefore, it was crucial to regulate strain in the semipolar superlattice layer with an appropriate InGaN layer thickness to obtain high-quality semipolar (11$\bar{2}$2) InGaN/GaN superlattice relaxed templates. The results demonstrated that the strain relaxation in the superlattice layers was beneficial to improve the surface microtopography and increase the indium incorporation concentration in the InGaN/GaN superlattice layers. The research in this paper will be helpful for the comprehension of strain in semipolar (11$\bar{2}$2) superlattice layers, the design of semipolar relaxed templates and growing the high-quality relaxed InGaN/GaN superlattice templates for the fabrication of high-performance semipolar GaN-based devices.

## Figures and Tables

**Table 1:** The parameters of the superlattice templates of Sample A, Sample B and Sample C.

In${}_{x}$Ga${}_{1-x}$N/GaN Templates	Period	T ${}_{\mathrm{SL}}$(nm)	t(InGaN)/t(GaN) (nm)	δ along [$\bar{11}$23] ${(}^{\circ })$	MDs Spacing (nm)	$\rho_{\mathrm{MDs}}$ (cm ${}^{-1})$
Sample A	10	7.3	3.5/3.8	0.11	141	7$\;\times \;10^{4}$
Sample B	10	11.2	7.4/3.8	0.22	71	1$\;\times \;10^{5}$
Sample C	10	15.1	11.3/3.8	0.62	25	4$\;\times \;10^{5}$

**Table 2:** Lattice parameters of the GaN underlayers and the superlattice structures of Sample A, Sample B and Sample C.

	hk.l	d${}_{\mathrm{GaN}}$(Å)	d${}_{{\mathrm{SL}}_{0}}$(Å)	a${}_{\mathrm{GaN}}$(Å)	c${}_{\mathrm{GaN}}$(Å)	γ ${}_{\mathrm{GaN}}{(}^{\circ })$	a${}_{{\mathrm{SL}}_{0}}$(Å)	c${}_{{\mathrm{SL}}_{0}}$(Å)	γ ${}_{{\mathrm{SL}}_{0}}{(}^{\circ })$
Sample A	(11$\bar{2}$2)	1.3506	1.3613	3.2050	5.0591	120.21	3.2218	5.0945	119.60
	(11$\bar{2}$0)	1.5973	1.6107						
	(10$\bar{1}$1)	2.4421	–						
	(1$\bar{1}$00)	2.7696	2.7841						
Sample B	(11$\bar{2}$2)	1.3506	1.3652	3.1941	5.0447	119.91	3.2161	5.1199	119.46
	(11$\bar{2}$0)	1.5992	1.6139						
	(10$\bar{1}$1)	2.4374	2.4631						
	(1$\bar{1}$00)	2.7687	2.7878						
Sample C	(11$\bar{2}$2)	1.3499	1.3652	3.2036	5.0354	120.11	3.2278	5.1145	119.71
	(11$\bar{2}$0)	1.5992	1.6145						
	(10$\bar{1}$1)	2.4485	2.4745						
	(1$\bar{1}$00)	2.7714	2.7923						

**Table 3:** Calculated strain in the semipolar (11$\bar{2}$2) superlattice templates.

	In-Plane Strain		Out-of Plane Strain
	ε${}_{\left[\bar{11}23\right]}$ $\times \;10^{3}$	ε${}_{\left[1\bar{1}00\right]}$ $\times \;10^{3}$	ε${}_{\left(11\bar{2}2\right)}$ $\times \;10^{3}$
Sample A	7.7	∼0	8
Sample B	11.9	∼0	10.8
Sample C	12.5	1.4	11.3

## Data Availability

The data that support the findings of this study are available from the leading author, W.L., upon reasonable request.
